# Supplementary material for: Host autophagy machinery is diverted to the pathogen interface to mediate focal defense responses against the Irish potato famine pathogen
Source: eLife. 2018 Jun 22;7:e37476. doi: 10.7554/eLife.37476 (PMC6029844; doi:10.7554/eLife.37476)
Supplement: Supplementary file 1. [file elife-37476-supp1.docx]

**Supplementary File 1. Primers used in this study**

| **Name** | **Sequence (5’- 3’)** |
| --- | --- |
| GA_35s_F | CGTTCCAACCACGTCTTCAAAGCAAG |
| Cterm_BFP_Prom_R | GTTCTCCTTAATCAGCTCGCTCATTGATCCAAATTCTCCAGATCCAGCAGC |
| Cterm_BFP_F | GATCTGGAGAATTTGGATCAATGAGCGAGCTGATTAAGGAGAAC |
| Cterm_BFP_R | TCTAGCATGGCCGCGGGATTTAGTGCCCCAGTTTGCTAGG |
| GA_RD54_F | CTGGATCTGGAGAATTTGATGTTGGTCCCTCTTGGCT |
| GA_RD54_R | TAGCATGGCCGCGGGATTTACACAATTTCCCAGTCG |
| GA_LIR2_R | TAGCATGGCCGCGGGATTTAAGCAATTTCCGCGTCG |
| GA_Joka2_BFP_F | CAGGCGGCCGCACTAGTGATATGGCTATGGAGTCATCTATTGTGATCAAGG |
| GA_Joka2_BFP_R | GCAGATCCAGCAGATCCGATCTGCTCTCCAGCAATAAGATCCATCACAAC |
| GA_Joka2^Δ1-487^_F | CAGGCGGCCGCACTAGTGATGGATCGTCCTCTAACTTACCCGAATCCCTCA |
| GA_ATG9_F | CAGGCGGCCGCACTAGTGATGATGTTTGGTGGACAAAAAGG |
| GA_ATG9_R | GCAGATCCAGCAGATCCGATAACAAGATCTGAAGGATCTAACTTTCT |
| hpATG9_F | ACCAGGTCTCAGGAGAGGGTTGCTTGATGATGTGC |
| hpATG9_R | ACCAGGTCTCATCGTATTCCACAGCATCCATTCCAC |
| hpATG4_F | ACCAGGTCTCAGGAGGAACCAGGACCAAGCAGTAAC |
| hpATG4_R | ACCAGGTCTCATCGTTACTTTTGGTCATGCGGCTG |
| hpGUS_F | ACCAGGTCTCAGGAGTAAAGAGCTGATAGCGCGTGAC |
| hpGUS_R | ACCAGGTCTCATCGTTGTCGGTGTACATTGAGTGCAG |
| RT_ATG4_F | CGTTGGTTCTTGGACTTGAC |
| RT_ATG4_R | TTATCGTAAACAAAGGAGCACC |
| RT_ATG9A_F | CCCAATGGAGTGCGCAT |
| RT_ATG9A_R | ACGAAATAAACTTTGCGACGAC |
| RT_ATG9B_F | GGCCCTAATGGAGTGCATAG |
| RT_ATG9B_R | CAAAAGAAATGAACTTGCGATGAT |
